# Preparation of human amniotic membrane for transplantation in different application areas

**DOI:** 10.3389/frtra.2023.1152068

**Published:** 2023-05-05

**Authors:** Nicola Hofmann, Hans-Oliver Rennekampff, Anna Katharina Salz, Martin Börgel

**Affiliations:** ^1^ German Society for Tissue Transplantation (DGFG) gGmbH, Hannover, Germany; ^2^ Klinik für Plastische Chirurgie, Hand- und Verbrennungschirurgie, Rhein-Maas Klinikum GmbH, Würselen, Germany

**Keywords:** human amniotic membrane, preparation, preservation, application, characteristics

## Abstract

The human amniotic membrane (hAM) is the inner layer of the placenta and plays protective and nutritional roles for the fetus during pregnancy. It contains multiple growth factors and proteins that mediate unique regenerative properties and enhance wound healing in tissue regeneration. Due to these characteristics hAM has been successfully utilized in ophthalmology for many decades. This material has also found application in a variety of additional therapeutic areas. Particularly noteworthy are the extraordinary effects in the healing of chronic wounds and in the treatment of burns. But hAM has also been used successfully in gynecology, oral medicine, and plastic surgery and as a scaffold for *in vitro* cell culture approaches. This review aims to summarize the different graft preparation, preservation and storage techniques that are used and to present advantages and disadvantages of these methods. It shows the characteristics of the hAM according to the processing and storage methods used. The paper provides an overview of the currently mainly used application areas and raises new application possibilities. In addition, further preparation types like extracts, homogenates, and the resulting treatment alternatives are described.

## 1. Introduction

**Figure 1:**
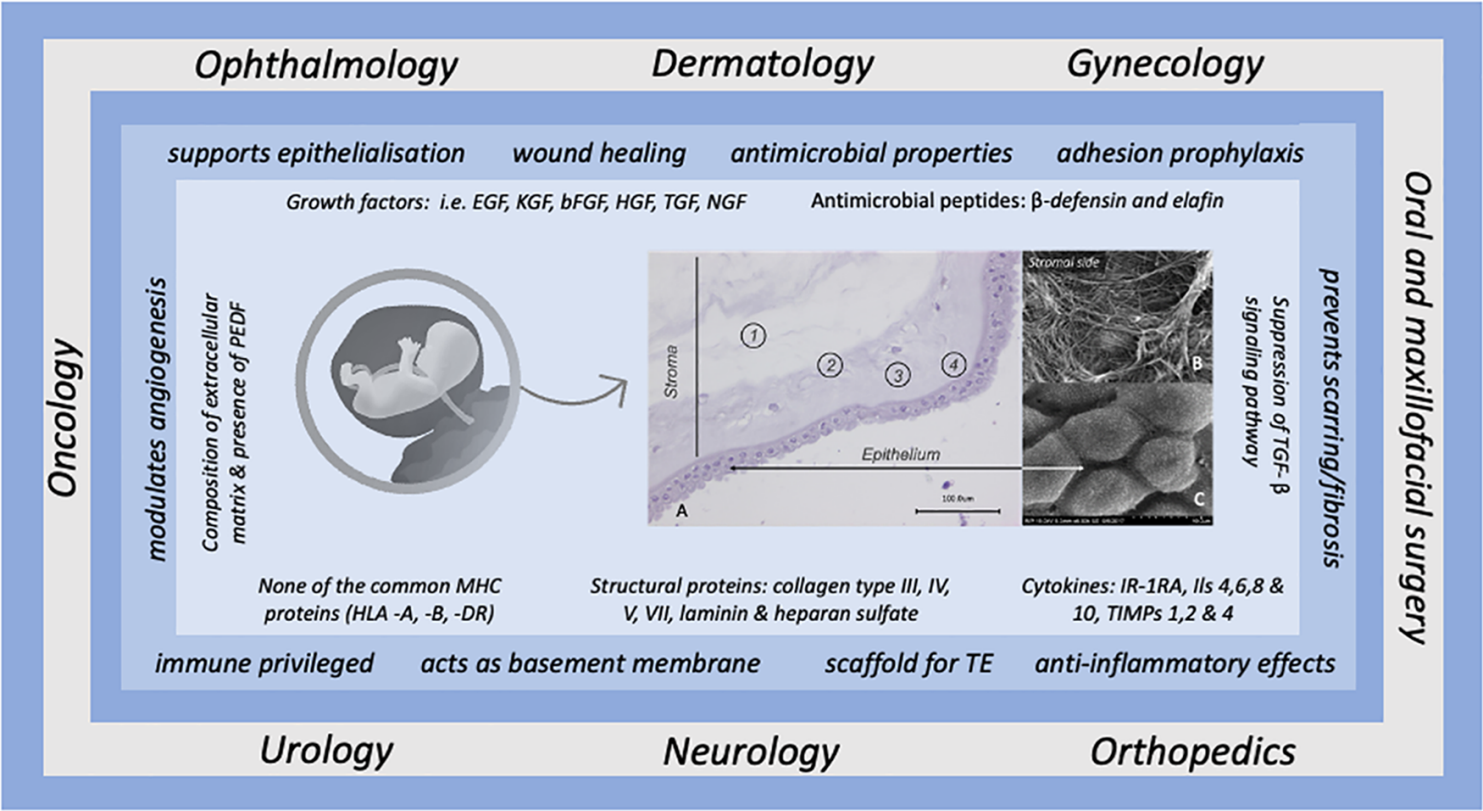
Overview of the most common applications of human amniotic membrane in conjunction with the beneficial effects attributed to it, based on the underlying biological factors. The histological illustration (left, **A**) shows the morphology of the hAM, the numbers indicate the individual structural elements of the hAM. 1 = Spongy layer, 2 = Fibroblast layer, 3 = Compact layer, 4 = Basement membrane. SEM images (right) display the surfaces (**B**) stromal side, (**C**) epithelial side at 5000x magnification (from Pogozhykh et al. (1). Pigment epithelial-derived factor (PEDF) is a non-inhibitory serpin with neuroprotective and antiangiogenic action (Tombran-Tink) (2)

The human amniotic membrane (hAM) is the inner layer of the placenta and plays protective and nutritional roles for the fetus during pregnancy. The hAM is a relatively simple tissue consisting of an epithelium and a stroma which in turn can be divided in the basement membrane and a compact, fibroblast and spongy layer, respectively (Figure 1). Its characteristics and properties make it ideal for use in many medical fields. For example, no or low amounts of HLA antigens (A, B, C, DR) are expressed by the cellular components (3, 4), so that hAM can be described as non-immunogenic (5, 6), and it is not rejected after transplantation (7–9), even after experimental xenotransplantation (10). This property is not too surprising since the human amniotic membrane is located during pregnancy between two individuals with their divergent immune systems (11). Antimicrobial (12), anti-inflammatory, and antiangiogenic properties have been demonstrated (13–16). The anti-fibrotic activity helps to prevent scarring, another important property that has made the use of amniotic membrane valuable in medical therapy (17–19).

In addition, amniotic membrane stimulates cell migration and proliferation, which are crucial for wound healing. Growth promotion is mainly mediated by growth factors such as epidermal growth factor (EGF), keratinocyte growth factor (KGF), hepatocyte growth factor (HGF) and fibroblast Growth Factor (bFGF) (20).

Due to its diverse characteristics hAM has been successfully utilized in ophthalmology for many decades, but also found application in additional therapeutic areas, such as healing of chronic wounds and in the treatment of burns, as well as in gynecology, oral medicine, and plastic surgery. A recent review provides a comprehensive overview of the different therapeutic targets and the mechanisms of action involved (21). Figure 1 summarizes the most described functions of hAM in connection to their potential mode of action in different fields of medical applications (22, 23).

To utilize amniotic membrane for these therapeutic purposes without risk and with the most benefit for the patient it is necessary to apply the best possible ways of collection, processing and storage while preserving their necessary effective structures. The review aims to give an overview of the possible processing methods involved in human amniotic membrane preparation, storage, preservation, and the available data regarding the characteristics of the membrane prepared by each technique.

## 2. Preparation and preservation techniques of hAM

The human placenta is typically obtained during a planned cesarean delivery under sterile surgical conditions. The donors provide their informed consent and are tested for various infectious diseases (e.g., HIV, HCV, HBV, Treponema pallidum). This ensures a low risk of disease transmission, a low bioburden and a minimal risk of contamination right from the start. Usually, this is followed by cooled transport to a specialized tissue bank, where the placenta is processed.

Prior to processing, the tissue is examined for specific conditions that would exclude hAM donation following the European guide to the quality and safety of tissues and cells for human application (24), such as premature rupturing of membranes, malformation of the fetus, or presence of endometritis or meconium ileus. Irrespective of the further procedure, the preparation starts with cleaning the placenta of adherent blood residues by rinsing with physiological solution. Then, the amniotic membrane is separated from the placenta and the chorionic part.

Depending on how the hAM is stored eventually, incubation in antibiotic-containing solution follows for varying lengths of time, commonly at least 1 h and up to 24 h. Most processing methods use a carrier material, e.g., a nitrocellulose membrane, in the further course to keep the thin hAM manageable. In this way different sizes can be cut depending on the purpose for which the hAM is to be used. For ophthalmological applications, pieces of 2 cm × 2 cm are mostly sufficient; in dermatological wound healing, the areas must be larger (25).

The further procedure is determined by the type of preservation that is to be applied. Table 1 provides an overview about the main techniques that are used: fresh or deep frozen, cryopreserved, and dried, with heat-dried, air-dried and freeze-dried/lyophilized being common. Glycerol-preserved hAM can also be found, as well as mixed forms, some of which are patented and therefore not disclosed.

**Table 1:** Overview about the main techniques that are used to prepare and sterilize human amniotic membrane for medical application.

Preparation/preservation method	Desinfection/sterilization method	Storage condition/time	Product properties	Viable cells	Clinical application/performance	Advantage	Disadvantage	References	Comment
Fresh	Soaking in antimicrobial solution possible	Cooled for hours up to few days	Native characteristics	Yes	Effective (i.e., in ophthalmology, for burns, nerve repair, gynecology, gastric ulcer), but not better than fresh-frozen hAM	Highest conformity with physiological properties	Sterility questionable, as no possibility for screening in advance, short shelf life	(26–31)	Fr$esh hAM can cause some immune reactions or inflammatory responses (32, 33)
Fresh-frozen on carrier w/o medium or CPA	Soaking in antimicrobial solution	Frozen −80°C for max. 2 years	Matrix structure maintained, factors preserved	No	Wound repair and wound healing in Ophthalmology, Wound healing disorders, Burns	Simple process, good preservation of matrix structure and factor content. 2-fold freezing possible without quality loss. Applicable without additional steps by the user	Limited shelf life, Freezing equipment neccessary	(34–38)	
	and/or irradiation		Noticeable changes in structure but continuity of BM preserved		Possibly some functional loss	Certified sterility	Changes in structure and factor content	(39)	
	or peracetic acid (PAA)		Changes in structure but continuity of BM preserved			Sterile		(39)	
Frozen, protected w medium containing CPA	Soaking in antimicrobial solution	Frozen −80°C for max. 2 years, −28°C possible, but shorter shelf life (8 month)	Matrix structure maintained, factors preserved	Possibly, but low amount	Wound repair and wound healing in Ophthalmology, Wound healing disorders, Burns	Good preservation of matrix structure and factor content. Possibly few viable cells	Limited shelf life, freezing equipment neccessary. Somewhat higher effort with process. Pretreatment (rinsing) necessary at the user's site	(40–43)	Storage at −28°C possible but shelf life shorter (44)
Cryopreserved w medium containing CPA, controlled rate freezing	Soaking in antimicrobial solution	Frozen below −140°C, in ultralow freezer, in gas phase of LN2 or in liquid LN2, basically unlimited (min. 5 years)	Matrix structure maintained, factors preserved with viable cells possible	Verified by different groups	Wound repair and wound healing in Ophthalmology, Wound healing disorders, Burns	Good preservation of matrix structure and factor content. Optimised cryopreservation protocol preserves cell viability, basically unlimited shelf life	Complex freezing equipment neccessary. Higher effort with process. Logistically more difficult to manage. Pretreatment (rinsing) necessary at the user's site	(43, 45, 46)	Antifibrotic activity maintained (47); supports chondrocyte proliferation (48) DMSO/Glycerol as CPA mostly equivalent
Heat-dried	Soaking in Antimicrobial solution	Room temperature, up to 5 years	Matrix structure with changes maintained, factors in reduced content preserved	No	Dressing/Wound covering in Ophthalmology, Wound care, Burns	Easy storage and logistically easy to handle. If irradiated, safely sterile. Longer shelf life	Loss of structural integrity and factor content. Depending on the application, pretreatment by the user may be necessary (moistening)	(49)	
	and/or irradiation		additional changes to be expected			Certified sterility	Further reduced structural quality and factor content		
Air-dried	Soaking in antimicrobial solution and/or irradiation	Room temperature, up to 5 years	Matrix structure with changes maintained, factors in reduced content preserved	No	Dressing/Wound covering in Ophthalmology, Wound care, Burns	Easy processing, easy storage and logistically easy to handle. If irradiated, safely sterile. Longer shelf life	Loss of structural integrity and factor content. Depending on the application, pretreatment by the user may be necessary (moistening)		
	and/or irradiation		Noticeable changes in structure but continuity of BM preserved		Barrier function maintained	Certified sterility	Further reduced structural quality and factor content	(50)	
	or peracetic acid (PAA)		Histological changes in structure but continuity of BM preserved			Sterile		(37, 39, 51)	
Lyophylized	Soaking in antimicrobial solution	Room temperature, up to 5 years	Matrix structure with changes maintained, factors in reduced content preserved	If optimized process using lyoprotectants established, possibly yes	Dressing/Wound covering in Ophthalmology, Wound care, Burns	Easy storage and logistically easy to handle. Longer shelf life	More complex process with special equipment required. Certain up to significant loss of structural integrity and factor content. Depending on the application, pretreatment by the user may be necessary (moistening)	(35, 43, 52)	
	and/or irradiation		Noticeable additional changes in structure and factor content	If irradiated: No	Possibly some functional loss	Certified sterility	Potentially further reduced structural quality and factor content, no viable cells	(37, 42, 53–56)	
	or peracetic acid (PAA)		Minor additional changes in structure, continuity of BM preserved			Sterile		(39)	Quality of wound dressing: continuity of amniotic epithelium
Glycerolized	Glycerol serves as a disinfectant itself, may be additionally soaked in antimicrobial solution	4°C, up to 2 years	Matrix structure changed, membrane factors in reduced content preserved	No	Ophthalmology, Wound care, Burns	Storage relatively easy and logistically simple to handle. Longer shelf life. Low cost, technical feasible	Histologically markedly altered matrix structure, altered thickness of the membrane. Strongly reduced factor content. Pretreatment (rinsing) necessary at the user's site	(57–59)	Storage in 98% glycerol (60)
	or additionally irradiated		Noticeable additional changes in structure, possibly some functional loss,		As substrate for cell culture	Certified sterility		(61)	
	or use of peracetic acid (PAA)		Changes in structure but continuity of BM preserved			Sterile		(39)	
Decellularized	Decellularization by enzyme-, detergent- or mechanical procedures contribute to desinfection, may be additionally soaked in antimicrobial solution and/or irradidated	Decellularization process independent of the storage method: frozen, dried or glycerolized possible. No information about shelf life	ECM structure with changes maintained, few factors, if at all in strongly reduced content present	No	Mainly used as scaffold for *ex vivo* cell culture/tissue engineering. Efficient substrate for LSC expansion	Safe system to provide adequate structural and biomechanical microarchitecture. No foreign body reaction	Markedly altered matrix structure, altered thickness of the membrane. Strongly reduced factor content.	(62–64)	
	or peracetic acid (PAA)		Major components of BM maintained		Substrate for the ex-vivo expansion of limbal epithelial cells, support the attachment and proliferation of primary human fibroblasts and keratinocytes	Sterile		(65, 66)	
					Decellularized and lyophilized: promising results for guided bone regeneration			(67)	

BM, basement membrane.

For *fresh-frozen* storage, the hAM is frozen on the carrier material at −80°C without further additives. *Deep-frozen* storage at −80°C is also possible: cryoprotected in a solution containing freeze-protective agents such as glycerol or dimethyl sulfoxide (DMSO), typically in a concentration of 5%–10% or in culture medium such as RPMI or physiological solutions without further protection. Samples can be stored for up to 2 years, but a freezer capable of holding temperatures of −80°C is required. Although it has been shown that storage at −20°C to −28°C is possible with a shorter storage time (44, 68). After thawing, tissues must be used within 6 h.

Glycerol or DMSO in this concentration are also used as cryoprotective agents (CPA) for *cryopreservation* of hAM. Cryopreservation, by definition, requires, in addition to using CPAs, a controlled freezing process by means of a controlled rate freezer to a deep-cold temperature (24). In addition to slow freezing, there is the possibility of vitrification, a process in which the aqueous phase is transformed directly to a glass phase (69). However, this has only been used experimentally so far. The final temperature is usually between −80°C to −140°C but can be as low as liquid nitrogen (LN_2_) temperature (−196°C). Subsequent storage should always be below the glass transition temperature of water, i.e., colder than −137°C to prevent further recrystallization that could lead to freezing damage. Ultralow freezers are used to reach about −152°C, alternatively storage is in the gas phase of LN_2_ at about −180°C or directly in LN_2_. In the liquid phase, however, it is necessary to seal the storage vessels so that no liquid nitrogen can penetrate, since it is only possible to produce sterile liquid nitrogen with great effort and there is otherwise a risk of contamination of the material. Cryopreservation allows longer storage. In principle, samples stored constantly below −137°C have an indefinite stability without further changes in quality, but usually 5 years shelf life is given for this condition. The disadvantage is the relatively complex and costly equipment needed for both the freezing process and long-term storage.

If the hAM is to be stored *dry*, it can be air-dried under a sterile class A workbench or heat-dried in an oven at about 40°C overnight after preparation. Alternatively, the tissue can be *lyophilized*, in which case a rapid freezing process to −80°C is followed by vacuum-drying in special devices. For this purpose, protective agents (LPA = lyoprotective agent) like trehalose or sucrose should be used (52, 56, 70), which on the one hand must protect against the damage caused by freezing, but additionally against the adverse effects of drying. Drying allows storage without special equipment at room temperature for a longer period which offers logistical advantages.

*Glycerolized* hAM is also used. For its production, the membrane is usually preserved in 85% glycerol and can thus be stored at 2°C–8°C. It has also been described that the glycerol concentration can be increased to 98% without compromising the clinical efficacy of hAM (60). The advantage is the simple storage with a relatively long shelf life of 2 years. However, grafts must be pretreated before use so that the patient is not affected by the glycerol.

Decellularization is a further option for processing hAM. Enzymes, detergents and/or mechanical procedures remove the cellular components. Although extracellular matrix structure is mainly preserved, it shows considerable changes (63). While ECM components like collagens I, II, IV, VI and VII, laminin-5, fibronectin, elastin and thrombospondin, remain present after most of the techniques, growth factors like TGF-a,-b1 and -b2 receptor, EGFR, KGF, bFGF, VEGF, and PDGF are only found when gentle procedures are used (43).

To make the use of hAM safe for the patient, it is essential to apply all possible measures that support sterility of the tissue. As described, this starts with the collection in a clean operation theatre and continues with the preparation under sterile conditions. While the use of cryogenic preservation procedures usually focuses on the use of antimicrobial substances, drying procedures are often followed by sterilization steps using irradiation with at least 25 kGy. Irradiation is also used for glycerolized hAM for sterilization purposes (71). In addition, glycerol itself has a disinfecting effect (59). Another possibility is the use of peracetic acid (PAA) for sterilization (39).

Most of the above-mentioned procedures are also used for hAM preparations which are commercially available, with the vast majority being freeze-dried variants. A comprehensive list of commercial hAM products can be found in a publication by Munoz-Torres et al. (21).

## 3. Characteristics of hAM in relation to the processing methods

Processing procedures have an impact on the properties of the human amniotic membrane (43).

**Figure 2:**
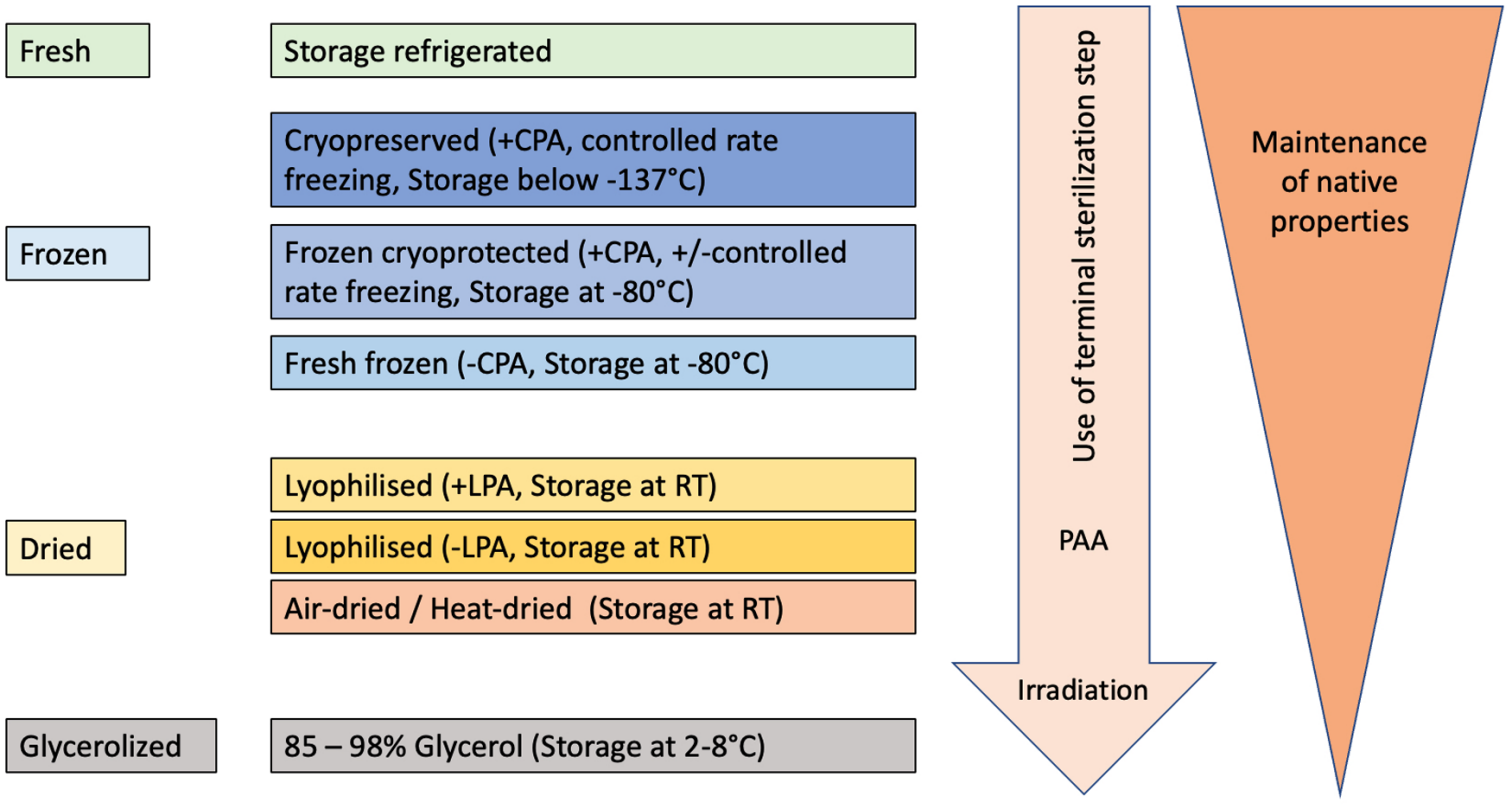
Schematic view of decreasing preservation of native properties of hAM depending on the degree of manipulation during processing, sterilization, and storage procedures.

The method used depends primarily on what impact on the biological material is desired or accepted. If for example the presence of viable cells in the hAM is irrelevant, stricter measures can be applied than if the vitality of the tissue is considered most important. Thus, the combination chosen must be weighed against which of the product's properties will ultimately be given the highest priority (Figure 2). For example, in ophthalmology the amniotic basement membrane facilitates migration and growth of epithelial cells, therefore promoting epithelialization. The avascular stroma of the hAM reduces fibrovascular ingrowth and abnormal neovascularization. And amniotic epithelium contains anti-inflammatory and growth factors beneficial to the treatment of inflammatory corneal diseases (72).

Irrespective of the preparation and preservation processes the overall structural properties of the hAM are largely preserved. For example, the architecture of the basement membrane (BM) is maintained (39), which is considered to be the most important element for the application in ophthalmology (18). The hAM's basement membrane contains Type IV collagen, laminin 1, laminin 5, and collagen VII like the cornea BM, and these components are important for epithelial adhesion and growth (34).

Especially the freezing/cryopreservation procedures do not significantly alter the histological appearance and architectural properties of hAM (37, 73). Tan et al. (74) confirmed by histochemical staining that the cryopreservation process did not noticeably change the tissue architecture nor collagen and glycosaminoglycan density. In addition, important other structural components like high molecular weight hyaluronic acid or heavy chain-HA complex (i.e., the HC–HA/PTX3 complex) appear to be preserved better by cryopreservation than by drying (34, 75). Furthermore, it was shown that even after a second freezing process, the structural integrity of the hAM is maintained (1) with morphologically intact, nonviable cells. While hAM can be used directly after fresh freezing, it must first be rinsed after CPA containing freezing/cryopreservation processes to remove the substances. It cannot be ruled out that this may result in the loss of factors that were retained during the actual preservation process.

However, differences appear in the thickness of the final product: while after drying the tissue is 20–30 μm thick, hAM thickness varied between 45 and 50 μm after glycerol preservation due to liquid deposit in the membrane (39).

Biomechanical characteristics of hAM described by Dadkhah-Tehrani et al. (23) seem also to be well preserved by freezing (1, 36): Tensile strength and Young's modulus were not influenced by frozen storage methods regardless of whether a CPA (glycerol) was used or the membrane was frozen without additives. This does not change even if the material is only stored at −28°C instead of −80°C (44). However, these parameters are significantly altered when the material is dried (76). This factor should be considered since elasticity, in addition to practical considerations during surgery, could also influence the adherence, proliferation and phenotype of cells (77).

It should be considered that mechanical properties also differ due to the placental region from which the hAM originates, since placental hAM was shown to be significantly stronger and more stretchable than their peripheral counterparts (78).

While the structure of the membrane is mostly preserved by the various processes, the effects on the protein level are more significant. Although there are already major interindividual differences in the amount and occurrence of individual factors isolated from hAM from different donor placentas (79, 80), the preservation techniques additionally change their concentration significantly (35, 53, 81, 82). Again, freezing processes do not have as much effect overall as drying or lyophilization methods (42, 83). However, optimization of the drying process using lyoprotective agents such as trehalose or raffinose can reduce the changes (52). It must be mentioned that different factors were examined in each of the various studies. Together with the circumstance of the above mentioned interindividual differences and additionally the different processing methods, consequently the results are not directly comparable. The largely consistent statement in literature is that the losses of the various factors investigated are lower in freezing than in drying processes or in glycerol. It is also uniformly described that final sterilization steps, especially by high irradiation doses, but also when PAA is used, have a negative effect on the respective proteins/cytokines under consideration (51). A combination of the procedures further reduces the levels (53). Moreover, it can be assumed from the individual studies that the diverse growth factors and cytokines are differently susceptible to the influences of the various methods due to the individual sensitivity of proteins to external conditions such as temperature and hydration status. For example, while TGF-ß levels seem to be relatively unaffected by the different treatment methods, EGF concentration was found to be significantly reduced, especially by irradiation (53).

The most drastic changes after preparation and preservation procedures are found in the presence of living cells in the final product. For all those techniques that are suboptimal for cell survival it cannot be assumed that viable cells are present to a significant amount following the procedure. This is especially true for the uncontrolled freezing procedures without CPA and the drying methods (84). For glycerol storage, it has also been shown that viability of cells is not preserved (58, 81).

In contrast with respect to the viability of the existing cells an optimized cryopreservation strategy with CPA and adjusted freezing rates is preferable. Up to 82% viable cells could be achieved (85). Differences occur in the final storage temperature where the highest survival rate was achieved at −196°C by Hettiarachchi et al. (45) but only 13%–18% reported by Hennerbichler et al. (58) with this procedure. Storage at −80°C, on the other hand, already considerably reduces the proportion of vital cells with increasing losses over storage time, as recrystallization processes continue to take place at this temperature, which affect the integrity of the cells. Conversely, the preservation of vital cells also has an influence on the factor content, since living cells continue to produce proteins. For example, the preparation method affects the angiogenic factor (AF) profile via the cell vitality (81). Nevertheless, the relevance of vital cells for clinical outcome has not yet been clarified (86). The multitude of published positive healing results achieved with material in which no living cells were present show that, as a rule, the vitality of the cells does not play a decisive role. Adds et al. (26) showed that application of fresh membrane, presumably with vital cells, did not achieve re-epithelialization of the cornea better than cryopreserved hAM. A similar conclusion was reached by Fenelon et al. (67), who found no difference between cryopreserved and fresh hAM for guided bone regeneration. In fact, the few adverse reactions described occurred when fresh membrane was used (32, 33), so it could be suggested that viable cells may not be advantageous for the most commonly used applications at present.

As described before, the highest priority must be given to the safety of the application to the treated patient. To achieve a sterile product, various sterilization processes are used.

Numerous preparation methods apply an antibiosis step to avoid contamination of the hAM with microorganisms. For rinsing with antimicrobial substances mostly streptomycin/penicillin mixtures in combination with the antimycotic amphotericin B are used (73). Recently, some antibiotics like gentamicin and ciprofloxacin have been shown to cause changes in the ultrastructure of the membrane (87) even though these do not seem to have any effect on clinical efficiency. However, rinsing will always affect the soluble protein content. It can therefore be expected that the duration of antibiosis will also reduce the quantity of proteins and thus alter the factor profile of the hAM.

It is agreed that the use of antimicrobial substances cannot be considered a terminal sterilization measure. Gamma irradiation or by electron-beam is most commonly used for this purpose. However, it is consistently reported that this type of treatment exerts the strongest influence on the properties of the amniotic membrane. This includes both the structure and the biomechanical characteristics associated with it. In addition, the proteins present in the membrane are significantly affected. Living cells cannot be detected after these procedures (88). In addition, the changes triggered by the irradiation add up to those already caused by the preparation and/or preservation process. However, it is described that the original antimicrobial properties of hAM (89) are maintained even after higher doses of gamma irradiation (90). The same authors describe a change in pH after irradiation, which could be particularly significant for the treatment of chronic wounds (91, 92). Nevertheless, the result is a guaranteed sterile product. This may be favorable especially for the use in open wounds (90).

In any case and independent of the way of processing the hAM will be subjected to a final product control for sterility before it can be released for use.

## 4. Currently predominantly used application areas for hAM

In this section the most prominent indications for hAM utilization are presented. Due to its special properties, new applications for hAM are constantly being developed, although the focus is increasingly shifting to the cells that can be isolated from birth associated tissues like amniotic membrane or umbilical cord (93).

### 4.1. Ophthalmology

Since 1940, when de Rötth (94) described the first amniotic membrane transplantation (AMT) to cover conjunctival defects, this therapy has become a routine procedure in ophthalmology. Thus, these application fields are described comprehensively:

• ocular surface disturbances caused by physical or chemical injuries, infections or systemic disorders may cause scarring of the conjunctiva or result in persistent ocular inflammation
• corneal ulceration with progressive thinning, descemetocoele and/or corneal perforation
• symptomatic bullous keratopathy, corneal disorders with associated limbal stem cell deficiency
• recurrence-free pterygium surgery
• other ocular surface diseases with large portions of bare sclera, such as dysplasia, tumors, scars and symblepharon
• corneoscleral melts and perforations
• in glaucoma treatment to reduce scarring in filtering surgery

[for detailed review see among others (18, 72, 95, 94)].

Among this the reconstruction of the corneal surface is one of the most common aims for AMT. In the majority of published studies, the amniotic membrane is used as a patch and is fixed to the ocular surface so that the corneal epithelium can grow over it.

In the case of deeper injuries or ulcers, inlay techniques are also used, whereby one or more membranes are placed in the cavity and, if necessary, covered with another hAM as an overlay.

There is controversy about the necessary orientation of the membrane; however, the chorionic/stromal side is usually placed toward the surface. It is assumed that the hAM acts mainly as a guide rail and basement membrane. This would explain why those different preparation, storage, and sterilization procedures, which have a profound influence on the factor content but preserve the matrix structure seem to have no negative effect on the clinical efficacy for healing corneal surface defects. Many clinical data are available for this application, which were achieved with hAM prepared according to all conceivable procedures.

For a long time, fixation on the corneal surface was done via a suture. Thus, most applications were limited to the one-time coverage of a defect. More recently, constructs like PROKERA® (97) and AmnioClip-plus (98, 99) have become available which allow the suture-free placing of the membrane on the ocular surface. For the use of these systems, data on the successful treatment of pathologies like Steven-Johnson Syndrome, toxic epidermal necrolysis or dry eye syndrome among others is existing (100–102). Here the guide rail function does not seem to play a major role. It can be hypothesized that the factors stored in the membrane trigger the clinical effect. In both systems, a freeze-storage procedure is used, so that it can be assumed that sufficient cytokines and growth factors are preserved in their native effectiveness.

With the development of cell therapy, hAM can be also used as a carrier for *ex vivo* pre-culturing of limbal stem cells (LSC) for reverse transplantation in limbal stem cell deficiency (LSCD) (103–105). For this purpose, it seems to be sufficient or even advantageous if the membrane merely forms the extracellular matrix for the cells. Therefore, the products that are more intensely processed can also be used. Decellularized or deepithelialized (denuded) hAM, for example, have proved successful for such applications as well as for tissue engineering (62, 69) but the preparation method to favor is the subject of controversy (43, 65, 106–108).

### 4.2. Dermatology

The use of amniotic membrane in dermatology began as early as 1910 when AMT was described as a skin substitute by Davis (109). A little later, Sabella and Stern (110, 111) used hAM to treat burns. Since then, AMT has become firmly established in dermatology and the number of clinical reports has been growing steadily (112, 113).

In dermatology hAM is used to treat chronic wounds that do poorly or not respond to other treatments, e.g., chronic ulcers of the lower leg or diabetic foot. Impaired wound healing is particularly common in patients with diabetes and is associated with serious complications such as ulceration, infection, and gangrene. Diabetic foot ulcers (DFU) can lead to costly complications like hospitalization, amputation, and increased mortality. Standard treatments (Standard of Care, SOC) for DFU and other chronic wounds often need to be supplemented with additional therapies to stimulate healing of recalcitrant wounds. For this objective, the application of amniotic membrane has already proven its benefit in many cases (114–120). Although cryopreserved hAM has also been used successfully in this regard (85, 121), most reports refer to dehydrated material, for which the actual manufacturing process used is usually not described. However, meta-analysis showed (113, 122) that the dehydrated material achieved statistically significant better wound closure than SOC alone (70%–97% healing with dhACM within 4–12 weeks compared to 15%–32% with SOC). Therefore, similar as in ophthalmology, the provision of the tissue as a scaffold for the growth of the patient's own cells can be seen as one mechanism of action, since it seems, that all hAM products with a well-preserved structure like dehydrated membrane are suitable. These types of products are listed by the FDA as “wound covering” or “dressings” to distinguish them from active agents.

More recently, research has also investigated other mechanisms of action of hAM in healing of chronic wounds. As could be expected, it appears that various growth factors and signaling molecules also interfere with the healing mechanism (123, 124). In this regard, it could be advantageous to use hAM material in which the protein profile with the corresponding factors is preserved. This was demonstrated for all freezing procedures.

Human amniotic membrane, frozen at −20°C to −80°C or cryopreserved via controlled process and stored at −180°C has also been successfully used to heal fistulas of different etiology (125–128). Because inflammatory processes play a major role in the persistence of fistulas (129), immunomodulatory properties of the hAM (130) seem to contribute to the modification of the inflammatory condition so that wound healing becomes possible (131).

Since immunomodulatory properties are primarily mediated by factors such as interleukin-10 (IL-10), transforming growth factor-b (TGF-b), hepatocyte growth factor (HGF), or prostaglandin E2 (PGE2), the success in the use of cryopreserved membrane may be due to the fact that freezing procedures sufficiently conserve these factors compared to dehydration (51).

The use of hAM has also been proven in the treatment of burns. It has been useful as a temporary epidermal substitute for covering wounds in the treatment of second-degree burns, and especially in children (49, 132, 133). Pain reduction, early wound drying and significantly accelerated wound healing with epithelialization are described as particular advantages (134–137). Additional positive effects seem to be a reduction of microorganisms, which is especially important in burns, as well as the promotion of neoangiogenesis (138). In addition to its use as a temporary skin substitute, the amniotic membrane is useable as a wound dressing for split-thickness skin-graft donor sites. Here the benefits are the improvement of the aesthetic result and the reduction of scarring. Basement membrane formation is accelerated, wound secretion is reduced and there is less itching, which increases patient satisfaction (139). The mechanism of action for these applications appears to be primarily based, again, on scaffold and basement membrane properties. Therapeutic success is described with all hAM products, regardless of how they were processed and sterilized (140–142). The antimicrobial property of particular interest for wound healing seems to be preserved by freezing as well as lyophilization (143).

In addition, a variety of approaches for this field of activity are described, in which hAM is used as a matrix for regenerative tissue engineering, including combined constructs (23, 41, 69).

### 4.3. Oral and maxillofacial surgery

#### 4.3.1. Amniotic membrane-assisted tissue regeneration in periodontal and oral surgery

Due to demographic change and the desire for a high quality of life into old age, dental health is increasingly becoming a focus of the population. In this context, dentistry is confronted with the widespread disease periodontitis and the associated long-term preservation of the natural chewing function. The reconstruction of the jawbone lost due to infection and the surrounding periodontium with its mucosa are fundamental measures for preserving the patient's own teeth. In the context of preparing an implant bed for reconstructions, the application of resorbable or non-resorbable membranes as a guide structure for functional tissue regeneration with simultaneous exclusion of inferior tissue is of central importance. Systematic reviews and meta-analyses have shown that guided tissue regeneration (GTR) procedures result in a significantly greater gain of attached gingiva than surgical procedures without the use of membranes (144, 145). At this point, hAM offers itself as an allogeneic biomaterial for the use of a natural GTR and guided bone regeneration (GBR) membrane. Its antimicrobial properties as well as its lack of immunogenicity offer infection prophylaxis in a physiologically bacterially contaminated surgical site (7, 146, 147). Furthermore, it fulfills important requirements for use as a barrier membrane due to its high tensile stability and anti-adhesive effect (148, 149). Previous studies on the use of hAM in periodontal and oral surgery confirm these results (150–153).

In hAM transplantation for GBR, not only a barrier function but also an induction of bone growth and regeneration could be demonstrated in both animal models and patients (154, 155). Furthermore, in a randomized controlled trial, hAM was shown to be equivalent to collagen membrane in GTR in covering gingival recessions (156). A reduction in periodontitis-induced gingival pockets and coverage of dental recessions were also successfully confirmed (157). Studies show excellent results for the use of hAM in the context of preprosthetic surgical procedures such as vestibuloplasty (158). The suitability as an intraoral wound dressing after local tissue excisions confirms the wide range of applications of hAM in periodontal and oral surgery (159, 160). Due to its intrinsic material properties, hAM represents an attractive alternative to established biomaterials and is able to demonstrate an alternative to 3rd generation drug- and growth factor-coated membranes [Kesting et al. (161), Review].

Other therapeutic options in this area include the management of medication-related osteonecrosis of the jaw (162–164) and mucosal defects, root coverage of gingival recession, and oronasal fistulae management (86).

#### 4.3.2. Adhesion prophylaxis in orbital fracture reconstruction

The human orbit has a volume of about 30 cm^3^, of which about a quarter is occupied by the bulb (165). It is formed by seven bones and has a bony thickness of only 0.3 mm in the area of the lamina papyracea. Frequently, orbital involvement occurs in the context of other midface fractures (>40%) (166). The clinical symptoms of an orbital fracture are manifold. An absolute emergency is the retrobulbar hematoma with the risk of permanent blindness. Surgical reconstruction of orbital fractures within the first 48 h after trauma significantly reduces the likelihood of persistent symptoms (167, 168). The goal of surgical orbital reconstruction is the unrestricted restoration of ocular function. The rate of persistent postoperative discomfort varies significantly in the literature from 0.53%–81% and regularly requires corrective surgery (169, 170). Known reasons for a limited postoperative result are on the one hand the fracture size, but also the choice of reconstruction material. In particular, the use of titanium implants and resorbable PDS®^1^ sheets is controversial regarding the induction of scarring adhesions.

The use of hAM is considered an innovative procedure for the therapy of orbital adhesions and strabismus (OAS) correction (171, 172) and has shown significant success both in animal experiments and in patients for adhesion prophylaxis of secondary motility disorders (149, 173, 174). Other properties such as pain modulating, angiogenic and anti-inflammatory effects make hAM an extremely promising biomaterial in anti-adhesive and reconstructive orbital surgery. The use of hAM can therefore also be helpful when using orbital implants and ocular prostheses after enucleation (175).

In view of the established use of the amniotic membrane in ophthalmology, a permanent extension of the indication of the amniotic membrane into the field of oral and maxillofacial surgery with a focus on the prophylaxis of the OAS after orbital fractures should be considered.

### 4.4. Gynecology

There are two main fields in gynecological applications of hAM: Asherman syndrome (intrauterine adhesions, IUA) and vaginoplasties. Intrauterine adhesions (IUAs) are a benign uterine disorder that results in intrauterine adhesions and scarring. The principle for the application of hAM to IUA is the use of a biologically active mechanical separator after hysteroscopic adhesiolysis (176). The reports show that fresh hAM graft improved the clinical outcome and reduced the recurrence of adhesion reformation (29, 177, 178) while meta-analyses of studies showed that dried or freeze-dried hAM increased menstrual blood volume but failed to improve the rates of intrauterine adhesion recurrence, pregnancy or spontaneous abortion (179–181).

Recent efforts are directed toward the use of human amniotic mesenchymal stromal cells for the management of IUAs by promoting endometrial regeneration and repair (182). The results of this investigation suggest that the divergent results of the above-mentioned studies are due to the use of differently pretreated products. For a successful treatment of IUAs with reduced recurrence rates active cells seem to be advantageous. Therefore, such membranes would be recommended for this application, which support presence of vital cells, e.g., via a controlled cryopreservation procedure.

Further, in various techniques of vaginoplasty, the use of hAM can be considered a safe and simple procedure with good functional results (183). The successful application of hAM in this field was already described in 1979 (184). Since then, various other groups have used this material with good clinical outcome (185–192). As both fresh and chemically processed and sterilized freeze-dried hAM were used, it can be assumed that the results are again based on the matrix effect of the membrane.

### 4.5. Urology

Every year, thousands of surgical procedures are performed to replace or repair ureters, urinary bladders or urethrae that are damaged through disease or trauma. In principle, the hAM is also suitable for this purpose (193), but it has been shown that the biomechanical properties of the thin membrane might not be sufficient for this intention. Therefore, the approaches in this area are focused on the use of composite material with chorion or other adjuvants (194–196), which shows promising results. An overview for such regenerative approaches is given by Nejad et al. (197).

Human amniotic membrane can improve tissue regeneration and functional outcome after radical prostatectomy (RP) due to the growth factors and unique immune tolerance. Preliminary studies showed the potential value of hAM in the reconstruction of the urinary tract and nerve protection during RP (198). Their results showed that dehydrated hAM could be considered as a suitable scaffold for faster improving vesicoureteral anastomosis (VUA) healing. Another clinical study was developed in 2020 by Barski et al. (199) to evaluate the efficacy and safety of hAM placed around the neurovascular bundle during RP for the treatment of the localized prostate cancer.

These applications are closely related to the use of hAM in Neurology below.

### 4.6. Neurology and neurosurgery

Multiple investigations—most of them, however, still in animal models—have highlighted hAMs role in preventing recurrence of perineural adhesions, reducing fibrosis, accelerating nerve repair, and improving nerve function. Thus, the amniotic membrane has ideal properties for treating peripheral nerve injuries (28, 41, 200). The authors conclude that the best would be a freeze-dried tissue containing the amnion and chorion layers in order to preserve all its growth factors and facilitate its handling and storage in the operating room. Another approach in this direction is to support the natural properties of the amniotic membrane by electrospun polycaprolactone (PCL) fibers (201). In particular for neurosurgery, several studies have shown its value for dural repair in both cranial and spine surgeries (202).

### 4.7. Orthopedics

Human amniotic membrane has been evaluated for the repair of tendon and ligament, attenuation of cartilage and joint space diseases, prevention of scarring and adhesion formation in spinal fusion procedures. It can prevent tendon adhesions after injury and reconstruction (41, 203). Products from all different processing methods are used with equal success. Furthermore, hAM as an injectable material is utilized to improve healing or manage pain in osteoarthritis (204, 205). For example, decellularized and dehydrated human amniotic/chorionic membrane is micronized and injected for knee osteoarthritis, chronic tendinosis or arthropathy (206–211). However, the particle size seems to play a role (212). For cartilage repair unchanged hypothermically stored membrane was used. The success of the treatment seems to depend on living cells in the product (213). Approaches that use cells or exosomes/extracellular vesicles (EVs) derived from them support that interpretation (21, 197, 214).

Over the last 20 years, there has been increasing interest in bone regeneration using hAM. Numerous reports show that hAM has a positive effect on bone healing as already mentioned in the oral surgery part. However, there is no consensus regarding the optimal usage strategies (215) as all kinds of preparation methods are used. Direct comparison between fresh, frozen, and decellularized freeze-dried membrane showed that decellularized and lyophilized hAM performed significantly better (67). This result would suggest that bone regeneration requires mainly the matrix of the membrane, rather than the content of growth factors. In contrast, studies are also known in which cells isolated from the amniotic membrane (amniotic-derived epithelial cells (AECs), and amniotic mesenchymal stromal cells (AMSCs)) associated with an appropriate scaffold seem to be ideal candidates for tissue engineering strategies applied to bone healing (41, 216).

### 4.8. Oncology

Human amniotic membrane has also generated increasing interest for applications in oncology (217, 218). Pro-apoptotic, anti-angiogenic as well as cell-cycle arrest, and immune-regulatory properties (219, 220) make the material a promising candidate for anticancer therapy. One approach is using the membrane as a matrix to line a cavity formed after surgery to prevent tumor regrowth by acting as a physical barrier and providing anti-angiogenic properties (221–224). However, most reports refer to the use of the factors present in the membrane or the cells that produce them (225, 226). Therefore, also the use of conditioned medium for the therapy of breast cancer or hepatocarcimoma cells (227, 228), of hAM homogenate in a model of bladder cancer (229) or extracts (218, 225) is described. The mode of action in these cases could be based on exosomes or EVs. Cryopreservation does not alter the anti-cancer activity of hAM (230), but the mode of homogenization seems to have an impact (229).

However, all results, especially if cells like placental mesenchymal stroma cells (MSCs) are used, must be considered with caution, as the opposite effects have also been described. For example, angiogenic properties may influence regrowth of tumors (130, 231–233). Since angiogenic factors appear to be reduced by freezing processes (81) it could therefore be useful to apply such processing methods for this purpose in order to make greater use of the anti-carcinogenic properties of hAM while avoiding opposite effects.

## 5. Further preparation types and application fields

The applications of human amniotic membrane are constantly expanding, so that further reports can be found, e.g., on the implantation of hAM on pancreatic anastomosis after pancreaticoduodenectomy (234), for palatal epithelial-connective tissue reconstruction (235) or for the treatment of open myelomeningocele (MMC) and lipomeningocele (LMC) (236). The hAM is used to improve the post-tonsillectomy recovery by reducing post-operative pain and bleeding and promoting the wound healing process (237) and in airway reconstruction following chondrosarcoma resection (238). Another approach is the treatment of liver fibrosis. For this purpose, the anti-fibrotic properties of hAM are useful, which were retained even after freezing (47).

As mentioned above for the individual application areas, in recent times newer application focus on more manipulated derivatives from hAM like extracts, homogenates or exosomes/EVs (239). Moreover, the membrane is used for tissue engineering purposes, i.e., combined with other materials such as hydrogel or fibers of different origin (23, 69, 240). For these purposes, often decellularized material is used, which can be produced by means of different techniques (43). A newer study investigates the effect of an injectable hydrogel generated from a decellularized amniotic membrane (dhAM-gel) on preventing the development of an intrauterine adhesion (241).

Some recent reports demonstrate the feasibility of producing vascular grafts by various techniques involving hAM for example by weaving yarn from the membrane or combine it with electrospun polycaprolactone (PCL)/silk fibroin (SF) (245, 246, 244). For this purpose, decellularized material is used, as the structural components are important.

To prepare an extract from human amniotic membrane (hAME), the membrane is usually obtained and purified as described above. The clean fresh, cryopreserved or dried hAM is then grounded generally using cryogenic temperature (LN_2_). The micronization can be done via mortars or e.g., cryo-mills, followed by an extraction, e.g., with physiological solution. After centrifugation, supernatants may be sterilized by filtering through a 0.2-μm pore size membrane (245, 246). Studies have shown that hAME used as eye drops (AMEED), supports proliferation and differentiation of corneal epithelial cells, enhances epithelial wound healing, and inhibits corneal neovascularization by supporting *in vivo* cultivation of limbal stem cells (244–249) whereby the extract is as efficient as AMT (250). Promising results are achieved for treatment of dry eye syndrome and Graft-versus-Host disease (251–253). The hAME can provide a new therapeutic strategy to modulate the bone density or calcification during bone regeneration by modifying osteogenic efficacy (254).

Alternatively, homogenates are used which in principle are prepared in the same way as extracts but without a centrifugation step. No sterile filtration step can be performed here, so sterility must be achieved prior to the processing by antibiotic treatment or by irradiation.

Homogenates of fresh and cryopreserved hAM show a strong antimicrobial effect, but it is important to choose the right storage condition to preserve this activity (224).

By processing into extracts or homogenates, the matrix structure is no longer preserved. However, the various process steps also have an influence on the product. For example, it has been demonstrated that the type of pretreating (frozen or freeze-dried) or grinding (pulverization vs. homogenization) changes the factor content (245, 255). Furthermore, the protein content seems lower in suspension/extracts in contrast to homogenates (256, 257) and could undergo an additional reduction by irradiation, but the remaining amount seems sufficient to achieve the desired healing effect, i.e., on human corneal epithelial cells (258, 259).

Injectable hAM particulates are successfully used for Osteoarthritis (260, 261) and the detrimental effect of homogenate on bladder cancer cells shows a promising option in oncologic therapy (229).

## 6. Discussion

For more than 100 years, birth-associated tissues such as human amniotic membrane have been used for clinical purposes. Despite early healing successes, the application could not initially find its way into routine use. It was only with the introduction of newer preparation and storage methods that guarantee a safe product that amniotic membrane transplantation became established in clinical practice in many medical disciplines. The aim of the present work is to summarize the most common processing and storage methods and applications currently in use, and to link these to the preferred properties of hAM.

Following our expectation, the literature has shown that all processes have an impact on the qualities of the finally used membrane. However, the described procedures and their influence on the properties of hAM are difficult to compare because on the one hand, the research groups used different methods for firstly the processing and preservation, secondly the factor/protein isolation and analysis and thirdly for evaluation and presentation of the results. Additionally, no uniform nomenclature is used: authors use identical terms for different procedures. In addition, the complete procedure is not always described. For example, it is usually left open whether fresh-frozen process without CPA is carried out in medium or liquid (e.g., buffer or salt solution) or dry. The term *cryopreservation* is used by most authors to describe all freezing procedures, independent of the freezing process performed, the added media/CPA used and the final storage temperature. The described changes in hAM properties due to the processing and preservation procedures can therefore not be assessed reliably or coherently. For example, Allen et al. (52) conclude that the optimized lyophilization process they used causes less damage to the tissue than cryopreservation. However, the process they describe for cryopreservation is an uncontrolled freezing in saline solution without protective measures, so any changes found as a result cannot be unexpected.

Similarly, for liquid derivatives of hAM, the terms *extract* and *homogenate* are used interchangeably, although the preparation of the two formulations is different. This also applies to *suspension* and *conditioned medium*.

The terms *viability* and *vitality* are also not used uniformly. Some authors seem to use it to refer to the remaining activity in the sense of the effectiveness of the hAM rather than referring to living cells. At the same time, studies show that vital cells do not necessarily need to be present for effectiveness of the treatment (86) unless a high factor content is essential, as living cells continue to produce proteins after transplantation (85).

In summary, all procedures presented in this review have influence on the properties and hence the possible way of usage of the amniotic membrane. Prior to a clinical application it should be carefully evaluated which properties of hAM are needed, such as matrix or guide rail functions, or factor release and immunomodulating activities by cells, respectively.

The huge number of publications shows that amniotic membrane has proven its effect and benefit in various fields of clinical applications, making it an essential treatment option for patient care.

## Footnotes

^1^PDS® sheets consist of resorbable polydioxanone, which is completely replaced by the patient's own body tissue after 180 days.
